# Autosomal Dominant Hypercholesterolemia: Needs for Early Diagnosis and Cascade Screening in the Tunisian Population

**DOI:** 10.2174/138920213804999200

**Published:** 2013-03

**Authors:** Awatef Jelassi, Mohamed Najah, Afef Slimani, Imen Jguirim, Mohamed Naceur Slimane, Mathilde Varret

**Affiliations:** 1 Research Unit of Genetic and Biologic Factors of Atherosclerosis, Faculty of Medicine, Monastir; Tunisia; 2 INSERM U698, CHU Xavier Bichat, Université Paris Denis Diderot, France

**Keywords:** Autosomal dominant hypercholesterolemia, Screening protocol, Molecular default.

## Abstract

Autosomal dominant hypercholesterolemia (ADH) is characterized by an isolated elevation of plasmatic low-density lipoprotein (LDL), which predisposes to premature coronary artery disease (CAD) and early death. ADH is largely due to mutations in the low-density lipoprotein receptor gene (*LDLR*), the apolipoprotein B-100 gene (*APOB*), or the proprotein convertase subtilisin/kexin type 9 (*PCSK9*). Early diagnosis and initiation of treatment can modify the disease progression and its outcomes. Therefore, cascade screening protocol with a combination of plasmatic lipid measurements and DNA testing is used to identify relatives of index cases with a clinical diagnosis of ADH. In Tunisia, an attenuated phenotypic expression of ADH was previously reported, indicating that the establishment of a special screening protocol is necessary for this population.

## INTRODUCTION

 Autosomal dominant hypercholesterolemia (ADH) was firstly reported by Carl Müller in 1938 with the study of families presenting tendon xanthomas and heart disease due to a hypercholesterolaemia dominantly inherited [1]. In the 1970’s, several studies in patients and cultured cells revealed a defect in the receptor for the low-density lipoprotein and culminated in the Nobel Prize for Brown and Goldstein for their work on the regulation of the cholesterol metabolism [2, 3]. ADH (OMIM # 143890) is one of the most frequent inherited disorders in humans, with a frequency of 1 in 500 for heterozygous in western populations [4]. In some populations, the frequency of heterozygous ADH is considerably higher because of founder effect. 

 A founder effect occurs when a subpopulation is formed through the immigration of a small number of “founder” subjects, followed by population expansion. If some of the founders had ADH, then genetic drift could lead to a high proportion of affected subjects who share specific mutations introduced by founders. Such founder effect was noted in French Canadians [5], South African Afrikaners [6], Jews [7], Indians [8], Christian Lebanese [9], Finns [10] and Tunisians [11].

 In Tunisia, the frequency was estimated at 1/165 for heterozygous, and beside the founder effect, the high birth rate and consanguinity marriage influenced this frequency [11].

 ADH is characterized by a selective increase of low-density lipoprotein (LDL) particles in plasma giving rise to tendon xanthomas, arcus cornea, and premature mortality from cardiovascular complications [12].

 ADH has proven to be genetically heterogeneous and is associated with defects in at least three different genes: *LDLR*, *APOB* and *PCSK9* genes. Other ADH-genes are still unidentified [13-16].

 In this review, we expose diagnosis protocol and molecular default causing ADH. We concluded on the necessity to establish special cutoff points for the Tunisian population.

## BIOLOGICAL, CLINICAL ASPECTS AND SCREENING PROTOCOLS FOR ADH

 Primary biological sign of ADH is elevated plasma cholesterol level [4, 17]. Plasma cholesterol level is largely modulated by different environmental factors. For these reasons, at least two measurements are required before a diagnosis of hypercholesterolemia can be made.

 Plasma cholesterol levels vary with age, sex, hormonal status, some acute illness, and are population dependant [4, 17, 18]. The cutoff level for diagnosis of hypercholesterolemia should thus ideally be age, sex, and population specific. 

 Heterozygous ADH patients usually have a twofold increase in total and LDL-cholesterol level. Homozygous ADH patients are characterized by an elevation of LDL-cholesterol often greater than 15 mmol/L (581 mg/dL) [19].

 Clinical signs for ADH are presence of tendon xanthomas and premature coronary heart disease (CHD). Homozygous patients commonly have tendon xanthomas before the age of 10 years, and, if untreated, they develop severe atherosclerosis and CHD within their third decade [19]. In heterozygous patients tendon xanthomas are common after 25-30 years old, and the onset of CHD is mostly before 55 years old [20].

 Diagnosis of ADH is mainly based on lipid levels, clinical signs, family history of dyslipidemia and/or premature CHD, and will be confirmed by genetic analysis. Three different diagnosis criteria were developed for ADH by the USMedPed (US Make early diagnosis Prevent early death) Program [18] (Table **1**), the Simon Broome Register Group in the United Kingdom [21, 22] (Table **2**), and the Dutch lipid Clinic Network [23] (Table **3**).

 Actually, in Tunisia, the Simon Broome Register criteria for ADH are mostly used to determine potential patient of ADH. Particularly the cutoff LDL-cholesterol of 4.9 mmol/L (190 mg/dL) is commonly used to determine heterozygote ADH patients. 

## CLINICAL AND BIOLOGICAL ASPECTS OF ADH IN TUNISIA AND CASCADE SCREENING

 Studies on ADH in Tunisia started in 1993 with the work of NM Slimane and coworkers. They estimated a high frequency of this disease for heterozygous (about 1/165). Beside they noted an attenuated phenotypic expression of ADH [11, 24]. 

 Indeed, the analysis of 91 ADH patients showed that the prevalence of CHD in Tunisian ADH heterozygous after 30 years old was 23.5% for men and 29.4% for women. All of them went through life without developing any tendon xanthomas (except one female aged 62). The mean total cholesterol level for heterozygous was 7.04 ± 1.40 mmol/L and was higher than the one reported in China (6.1 ± 1.2 mmol/L) [25], but lower than in Japan (8.8±2.0 mmol/L) [26], in UK (9.8± 1.7 mmol/L) [27], in Afrikaners (10.8±1.8 mmol/L) [28], or in Italy (8.49±1.66 mmol/L) [29]. The same observation was made concerning LDL-cholesterol levels.

 Concerning homozygous patients, xanthomas were present for all of them, CHD was present for 10% of them before 9 years old, for 71% between 10 and 19 years old. and for 100% above 20 years old. Therefore, CHD in Tunisian ADH homozygous appears to have a later onset than in other homozygous populations. Indeed, CHD occurs for 50% of the Afrikaners ADH homozygous patients before 9 years old. [6] and for 25% in Japan before 10 years old. [26]. Their mean life expectancy was 13 years old. compared with 17 years old in Japan (26) and 21 years old in Lebanon [29]. The mean total cholesterol level for homozygotes reported was 17.52±3.12 mmol/L [24], similar to those reported in other populations. 

 A recent study in Tunisia showed that 24% (9 out 38) of the ADH patients carrying an heterozygous mutation in the *LDLR* gene have a LDL-cholesterol level under the 60^th^ percentile of an age-and gender-matched reference population [30]. This discrepancy between the clinico/biological and molecular phenotype observed reveals the existence of factors that decrease the severity of the disease. In a previous study, we identified one of these factors as the traditional Tunisian diet which is enriched in polyunsaturated fats [11]. This type of diet has been shown to have long-term beneficial therapeutic effects by reducing the incidence of recurrent cardiovascular events. To conclude, 15 years after the first study in 1993 [11] similar characteristics of a mild phenotype of ADH in Tunisia was reported, particularly for heterozygous patients [24]. Thus, it appears clearly that despite the change in the diet habit to a more western diet, the Tunisian population still has the same mild clinical expression of ADH. According to these characteristics of the Tunisian population, the establishment of specific cutoff point seems to be necessary.

## MOLECULAR DEFECTS

 The known genetic bases of the ADH phenotype are mutations in the *LDLR*, *APOB*, or *PCSK9 *genes.

### In the LDL Receptor Gene (*LDLR*)

 The discovery of the LDL receptor and its defective function led to a great advance in the understanding of the pathophysiology of familial hypercholesterolemia (FH).

 The LDL receptor is produced in the endoplasmic reticulum (ER) where the 21 amino acid signal peptide is cleaved and the protein glycosylated to give rise to a mature receptor [31]. The 160kDa transmembrane receptor (glycoprotein of 839 amino acids) is present at the surface of most cell types and mediates endocytosis thus playing a pivotal role in cholesterol homeostasis [31].

 More than 1741 allelic variants have been identified in the *LDLR* gene and are distributed as presented in Fig. (**1**). All gene variants for *LDLR* are compiled online at two web sites: http://www.ucl.ac.uk/fh/ and http://http://www.umd.be/LDLR/.

 Functional *LDLR* mutations have been classified into five classes based on biosynthetic and functional studies of fibroblast cell [19, 32]. *Class 1* mutations are due to disruption of the promoter sequence, nonsense, frameshift or splicing mutations, all resulting in an absence of protein synthesis (null alleles). *Class 2* mutations, that primarily occur in the ligand binding and epidermal growth factor precursor domains, disrupt transport of the LDL receptor from the endoplasmic reticulum to the Golgi apparatus. *Class 3* mutations interfere with cell surface binding of the receptor to LDL, and these mutations are also primarily found in the ligand-binding and epidermal growth factor precursor domains. *Class 4* mutations appear in the cytoplasmic and membrane-spanning domains. They inhibit the clustering of the LDL receptor at the cell surface and the LDL internalization. *Class 5* mutations disrupt the recycling of the LDL receptor to the cell surface [19, 32].

 The first few defects in *LDLR* gene to be characterized were large deletions identified by southern blotting [33, 34]. Once amplification by PCR and direct automated sequencing of PCR products became possible the number of point mutation and minor deletions/insertions has greatly increased. The expanding use of multiplex ligation dependent probe amplification (MLPA), contributed to the evaluation of the exact contribution of major rearrangements. Recent sequencing into further intronic sequences has allowed identification of a large population of splice site mutations [35].

### In the Apolipoprotein B 100 Gene (*APOB*)

 The interaction between LDL and its receptor is fundamental for the regulation of plasma cholesterol in humans [31]. The only protein component of LDL is ApoB-100, which is the major ligand for the LDL receptor [36].

 ApoB-100, a large protein of 550 kDa, is encoded on chromosome 2 and has 26 exons. The binding region is rich in positively charged amino acids and interacts with the binding domains of the LDL receptor [20]. The domain of apoB-100 that interacts with the LDL receptor has been defined using several approaches. The proposed model of this binding region, comprising two clusters [A (31479-3367)] of basic amino acids that are linked through a disulfide bond between residues 3167 and 3297 [37, 38] has been further expanded though the discovery of the ADH causative mutation at residue 3500 [39, 40]. This has led to the general view that residues 3130-3630 are important for the binding of apoB-100 to the LDL receptor [41].

 With the development of immune-electron microscopy studies, it was demonstrated that normal receptor binding involves an interaction between Arginine 3500 and Tryptophan 4369 in the carboxy- tail of apoB100 [42]. 

 In contract to the numerous ADH causative mutations in the *LDLR *gene, only a very few mutations have been reported in the *APOB *gene. To date, 10 mutations in *APOB* gene were described. The most frequent one is p.Arg 3500Gln. This form of ADH, due to *APOB* gene mutations, was previously called FDB for Familial ligand-Defective apolipoprotein B (OMIM #144010).

 Compared with individual mutation in the *LDLR* gene, each of which is rare, the p.Arg3500Gln *APOB* mutation is common in Europe, where 2-5% of hyperholesterolemic are heterozygous carriers. The penetrance of the mutant *APOB* allele, however, is not 100%, so patients with familial ligand-defective apoB have a less-severe phenotypes than FH (Familial Hypercholesterolemia) patients with a *LDLR* mutation [43, 44].

### In the Proprotein Convertase Subtilisin Kexine Type 9 Gene

 The third locus causing ADH was identified to be a gene located at chromosome 1p32.3, and named proprotein convertase subtilisin kexine type 9 gene (*PCSK9*) [45].

*PCSK9* encodes the ninth member of the subtilisine-like protein convertase family (PCs). PCs are implicated in limited proteolysis of protein precursors going through the secretory pathway such as prohormones or precursors of neuropeptides [46]. 

 This gene comprises 12 exons transcribed into a complementary DNA that spans 3617 bp. The preproPCSK9 is synthesized as a 694 amino acid long, that undergoes autocatalytic cleavage between the prodomain and catalytic domain [47, 48]. The prodomain (~14 kDa) remains bound to the mature protein throughout the secretory pathway [49]. The mature PCSK9 and the associated prodomain both undergo tyrosine sulfation in the late Golgi complex before secretion [50]. The role of this post-translational modification in PCSK9 has not been defined [51].

 PCSK9 is now known to take part in the LDL receptor homeostasis [53,58-60]. Initially, three missense mutations in the *PCSK9* gene were identified in families with a clinical phenotype resembling FH and FDB: S127R, F216L [45] and D374Y [52]. Subsequently, additional missense mutations were identified in hypercholesterolemic subjects. 

 The observation that *PCSK9* mutations cause dominant hypercholesterolemia suggests that mutations confer a gain-of-function [45]. This hypothesis was confirmed by studies in which wild-type and mutant *PCSK9* (S127R, F216L) were expressed at high levels in the mice liver; hepatic LDL receptor fell dramatically in the mice receiving either the wild-type or mutant PCSK9 [50, 53]. No associated reduction in LDL receptor mRNA levels were observed. Thus overexpression of PCSK9, whether mutant or wild type, reduces the number of receptors through a post-transcriptional mechanism. However, the existence of a direct effect of PCSK9 on LDL receptor degradation has never been reported [54)].

 To confirm the hypothesis that loss-of-function mutant of PCSK9 would causes hypocholesterolemia, Cohen *et al.* [55] sequenced the coding region of *PCSK9* in individuals with low levels of plasma LDL-cholesterol (<5^th^ percentile). Surprisingly, one out of 50 African-Americans in the population had a nonsense mutation in *PCSK9* that lowered LDL-cholesterol levels by ~ 40% [55]. Subsequently, additional *PCSK9* mutations associated with a reduction in plasma levels of LDL-cholesterol have been found, including in-frame, and missense mutations [56,57].

 Until now, a total number of 101 unique variants were reported, covering the entire gene of *PCSK9*. [45, 50, 52, 56, 57] http://www.ucl.ac.uk/ldlr/Current/index.php?select_db=PCSK9]

 PCSK9, which interacts with the LDL receptor, is a promising therapeutic target for hypercholesterolemia and coronary artery disease. A clear link between PCSK9 and LDL-cholesterol is also observed in animal studies. Indeed PCSK9 knockout mice have decreased plasma LDL-cholesterol [58]. In non-human primates, PCSK9 knockdown by siRNA or inhibition by a monoclonal antibody also leads to decreased plasma LDL [59, 60].

 A recent study showed that antibody 1B20, which binds to PCSK9 with high affinity, disrupts the PCSK9-LDL receptor interaction, and inhibits the effect of PCSK9 on cellular LDL uptake [61]. Moreover, treatment with the 1B20 antiPCSK9 monoclonal antibody in mice and rhesus monkeys led to robust LDL-cholesterol lowering in plasma decreased liver PCSK9 and LDL mRNAs, and transient increases in total plasma levels of PCSK9 [61].

 To conclude, understanding the physiology of PCSK9 is important, and this protein has become a major new target for lipid lowering therapy.

### Other Possible Genes for ADH

 ADH has proven to be genetically heterogeneous and associated with defects in other still unknown genes. Indeed, in a Mexican population, no *PSCK9* mutations were found in one large ADH family that showed positive linkage to the 1.p34-32 locus. This indicates that genes other than *PCSK9* in the locus may be involved [13]. Marques-Pinheiro *et al.* [14] showed in a large family with ADH phenotype, but with no mutations in the three known genes, the implication of a fourth loci that was named *HCHOLA4.*This locus is located at 16q22.1 in a 7.89 Mb interval containing 154 genes.

 In the Chinese ADH population, after performing a genome-wide linkage analysis of a family pedigree without mutations in *LDLR*, *APOB* and *PCSK9* genes, a two suggestive linkage loci were identified on chromosome 3q25.1-26.1 and 21q22.3 [15].

 In ADH families from Spain, with no mutation in the known ADH-causing genes, Cenarro *et al.* demonstrated the implication of a new locus located in 8p.24-22 through linkage analyses [16].

 Finally, a Portuguese ADH study found only 48% of its total received cases with clinical diagnosis of ADH had genetic defects on *LDLR*, *APOB* or *PCSK9*, leaving the other 52% of ADH cases with possible undiscovered genes mutations [62].

 These studies confirm complex etiologies and suggest new genetic causal factors for the ADH disorder. 

## MOLECULAR DEFAULTS THAT CAUSE ADH IN TUNISIA

 In Tunisia, ADH is one of the most frequent genetic disorders with a frequency of 1/165 for heterozygous [11]. This population had a mild phenotype of ADH, in particularly for heterozygous carriers.

 Primary genetic studies were focused on *LDLR* gene. Recently, we started research on *PCSK9* gene variation. Concerning *APOB* gene, studies were realized to search for the p.Arg3500Gln mutation. Studies were carried on 102 patients from 19 unrelated ADH families. 

 Mutations identified in Tunisian ADH patients are presented in (Table **4**).

 In the *LDLR* gene, we identified 11 mutations in the different exons of the gene, from them 7 were novels. Mutations were nonsense, frame shift, missense and major rearrangement. The mutation p.Ser493ArgfsX44 in exon 10 appears to be the most frequent mutation. [11, 24, 30, 63, 64].

 Concerning the *PCSK9* gene, our team identified a novel missense mutation named c.520C>T (p.Pro174Ser) localized in exon 3. Study indicates that this new *PCSK9* variant is able to reduce the severity of FH, very probably acting as a loss-of-function variant. This finding should be confirmed by *in vitro* experiments [30]. 

 Moreover, four common polymorphisms of *PCSK9* were identified in a sample of 13 unrelated FH patients: L10, L11 p.474Val and pGlu670. Their frequencies were similar to those reported in previous study for different population [30].

 No APOB Arg3500Gln was identified in all patients genotyped until now. This observation was also noted for the Lebanese [65] and Moroccan [66] population.

 To conclude, the clinical expression of ADH in heterozygous patients is influenced by environmental factors as well as genetic factors in particular genes affecting lipoprotein metabolism such as *APOE*, *MTP*, *HL*, and *ABCA1* genes. In unrelated ADH patients, the plasma LDL-cholesterol level is influenced by *APOE*, *MTP* and *APOB* polymorphisms, and the plasma High Density Lipoprotein (HDL)-cholesterol level is influenced by *HL*, *FABP-2* and *LPL* polymorphisms [67]. The sequence analysis of these genes in Tunisian ADH patients may reveal genetic factors that are responsible of the mild clinical and biological phenotype of heterozygous ADH actually observed.

## CONCLUSION

 Special efforts are required to identify individuals with ADH in Tunisia as they are at high risk of premature coronary heart disease. The condition is seriously under diagnosed and the diagnosis is often made too late, in particular for heterozygous subjects, restricting the benefits of the treatments [68].

 These patients can be treated to lower their cholesterol levels, before the installation of CHD, and thus avoid the complications and early death. 

 The attenuated phenotype of ADH in Tunisia was demonstrated and the establishment of a special diagnosis protocol is essential for the Tunisian population.

## Figures and Tables

**Fig. (1):**
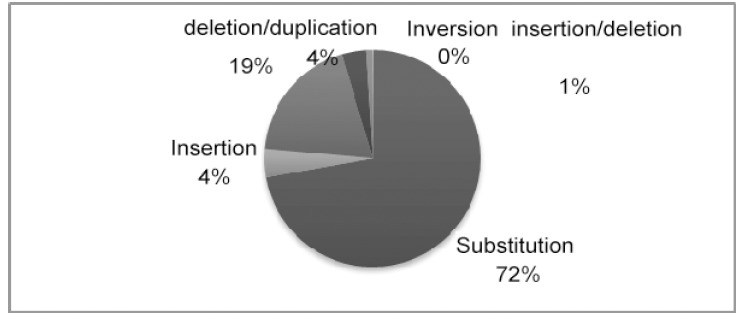
Distribution of molecular defects reported in the *LDLR* gene.

**Table 1.** US MedPed Program Diagnosis Criteria for Familial Hypercholesterolemia*

Total Cholesterol Cutpoints (mmol/L)				
	First-degree relative with FH	Second-degree relative with FH	Third-degree relative with FH	General Population
Age (years)				
<20	5.7	5.9	6.2	7.0
20- 29	6.2	6.5	6.7	7.5
30- 39	7.0	7.2	7.5	8.8
>40	7.5	7.8	8.0	9.3

Diagnosis: FH is diagnosed if total cholesterol levels exceed the cutpoint.

* Willams *et al.* Diagnosing heterozygous familial hypercholesterolemia using new practical criteria validated by molecular genetics. Am J Cardiol 1993; 72:171-6(8).

**Table 2.** Simon Broome Familial Hypercholesterolemia Register Diagnostic Criteria for ADH*

	*Description*
Criteria	
*a*	Total cholesterol concentration above 7.5 mmol/liter in adults or a total cholesterol concentration above 6.7 mmol/liter in children aged less than 16 years, or Low density lipoprotein cholesterol concentration above 4.9 mmol/liter in adults or above 4.0 mmol/liter in children
*b*	Tendinous xanthomata in the patient or a first-degree relative
*c*	DNA-based evidence of mutation in the *LDLR* or *APOB* gene
*d*	Family history of myocardial infarction before age 50 years in a second-degree relative or before age 60 years in a first degree relative
*e*	Family history of raised total cholesterol concentration above 7.5 mmol/liter in a first- or second-degree relative

A ‘definite’ ADH diagnosis requires either criteria *a* and *b* or criteria *c*

A’ propable’ ADH diagnosis requires either criteria *a* and *d* or criteria *a* and *e*

Risk of fatal coronary heart disease in familial hypercholesterolemia. Scientific Steering Committee on behalf of the Simon Broome Register Group. BMJ 1991; 303:893-6 (13).

Mortality in treated heterozygous familial hypercholesterolemia: implication for clinical management. Scientific Steering Committee on behalf of the simon Broome Register Group. Atherosclerosis 1999; 142:105-12 (14).

**Table 3.** Dutch Lip id Clinic Network Diagnosis Criteria for ADH*

	Points
Criteria	
**Family History** First-degree relative with known premature (men:<55 years; women:<60 years) coronary and vascular disease, or	
First-degree relative with known LDLC above the 95^th^ percentile	1
First-degree relative with tendinous xanthomata and/or arcus cornealis, or Children aged than 18 years with LDLC above the 95th percentile	2
**Clinical History**Patient with premature (men:<55 years; women<60 years) coronary artery disease	2
Patient with premature (men:<55 years; women<60 years) cerebral or peripheral vascular disease	1
**Physical Examination** Tendinous xanthomata	6
Arcus cornealis prior to age 45 years	4
**Cholesterol Levels (mmol/liter)** LDLC ≥ 8.5	8
LDLC 6.5-8.4	5
LDLC 5- 6.4	3
LDLC 4.0 – 4.9	1
**DNA Analysis** Functional mutation in the *LDLR* gene	8
**Diagnosis**(based on the total number of points obtained) A ‘definite’ ADH diagnosis requires more than 8 points A ‘probable’ ADH diagnosis requires 6-8 points A ‘possible’ ADH diagnosis requires 3-5 points	

World Health Organization, Familial hypercholesterolemia- report of a second WHO Consultation. Geneva, Switzerland: World Health Organisation, 1999. (WHO publication no. WHO/HGN/FH/CONS/99.2). (19)

**Table 4.** Molecular Defects Reported in Tunisian ADH Patients

Exon	cDNA Modification	Mutation at Peptide Level	Type of Mutation
**On* LDLR* Gene**
2_5*	g.11205052_11217736del12684	p.Gly23_Arg 271del	Major rearrangement
3	c.267C>G	p.Cys89Trp	Missense
4	c.443G>C	p.Cys148Ser	Missense
5	c.796G>A	p.Asp266Asn	Missense
5_6*	g.11216885_11219249 del2364	p.Ala231_Cys312del	Major rearrangement
7*	c.1027G>T	p.Gly343Cys	Missense
8-9*	c.1186+1G>A	p.Glu380_Gly396del	Splice site
10*	c.1477-1479del/insAGAGACA	p.Ser493ArgfsX44	Frame shift
12-13	c.1845+1G>A	?	Splice site
15*	c.2299delA	p.Met767CysfsX21	Frame shift
17*	c.2446A>T	p.Lys816X	Non sense
** On *PCSK9* Gene**
1	c.63_64 ins CTG	p.leu21 dup/Tri	Insertion
3*	c.520C>T	p.Pro174Ser	Missense
9	c.1420A>G	p.Ile474Val	Missense
12	c.2009G>A	p.Gly670Glu	Missense

* Mutations identified only in Tunisian patients.
